# Supplementary figures and images for: Direct-from-specimen microbial growth inhibition spectrums under antibiotic exposure and comparison to conventional antimicrobial susceptibility testing
Source: PLoS One. 2022 Feb 16;17(2):e0263868. doi: 10.1371/journal.pone.0263868 (PMC8849476; doi:10.1371/journal.pone.0263868)

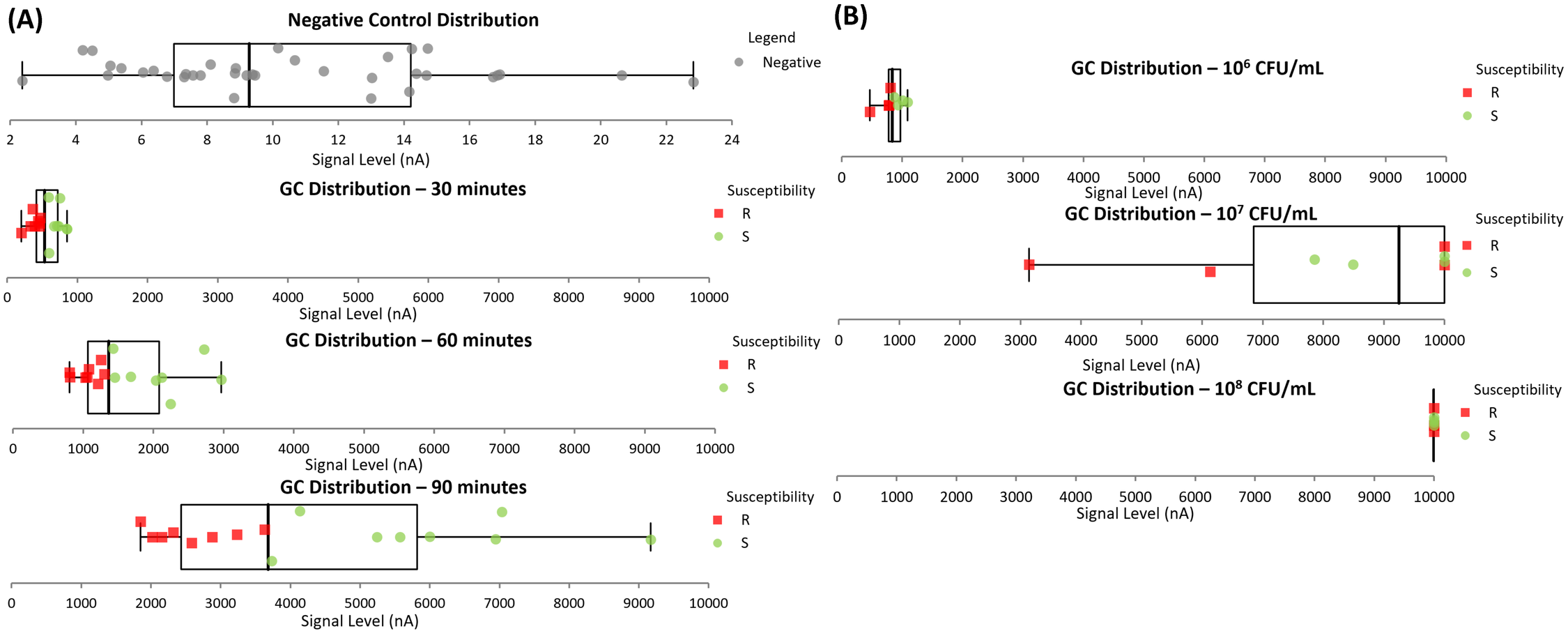

Supplement: S1 Fig — (A) Negative controls for Fig 3 and growth controls for Fig 3A and 3B, (B) Growth controls for Fig 3C. (TIF) [file pone.0263868.s001.tif]

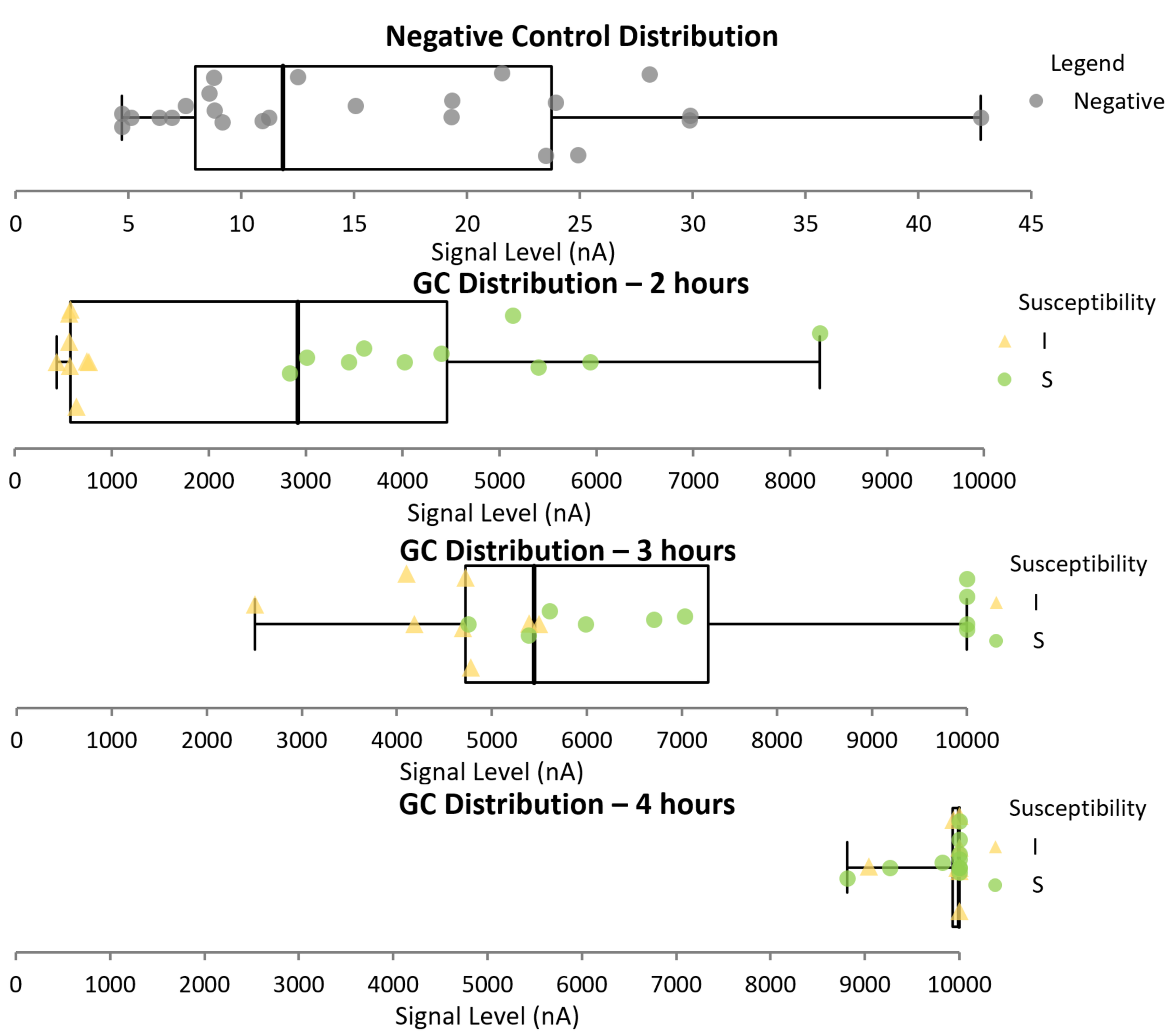

Supplement: S2 Fig — (TIF) [file pone.0263868.s002.tif]

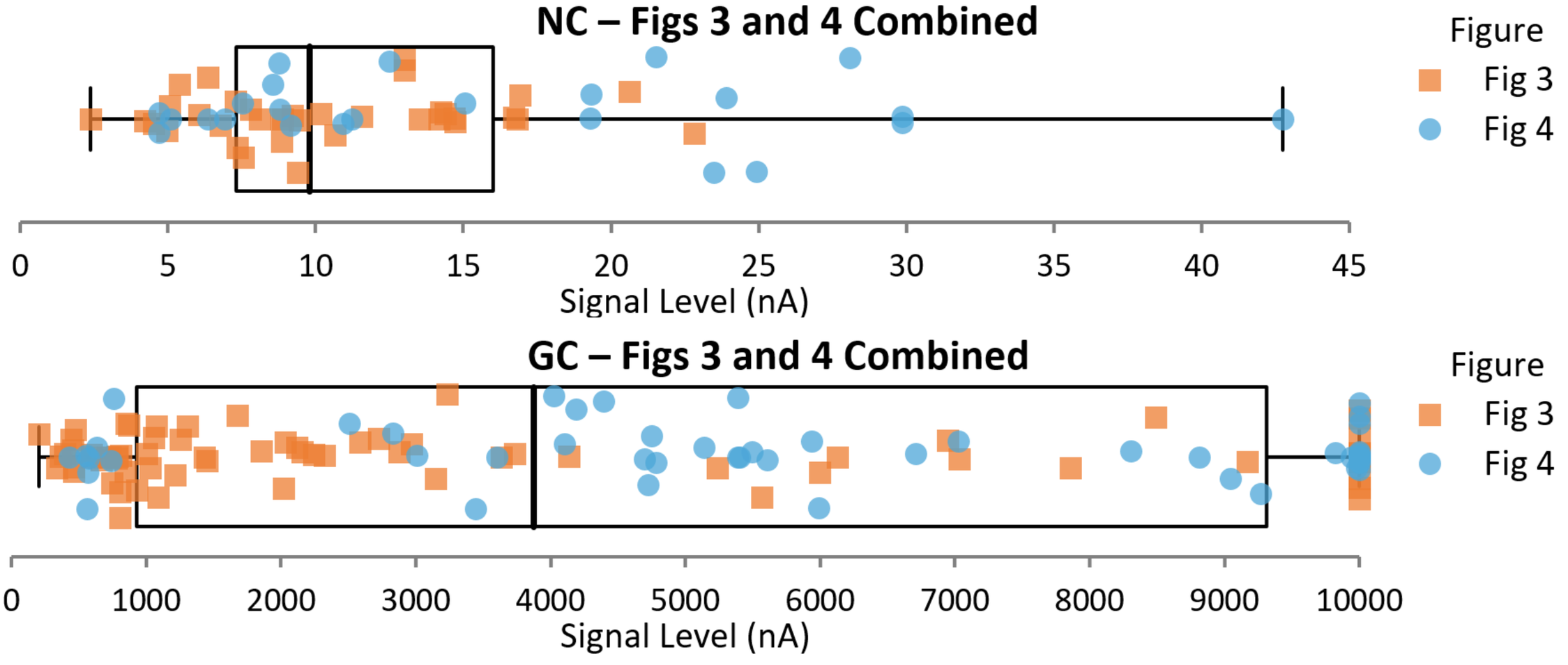

Supplement: S3 Fig — (TIF) [file pone.0263868.s003.tif]
