# Supplementary material for: Direct-from-specimen microbial growth inhibition spectrums under antibiotic exposure and comparison to conventional antimicrobial susceptibility testing
Source: PLoS One. 2022 Feb 16;17(2):e0263868. doi: 10.1371/journal.pone.0263868 (PMC8849476; doi:10.1371/journal.pone.0263868)
Supplement: S1 Table — On-scale strains in bold. (PDF) [file pone.0263868.s004.pdf]

S1 Table. Clinical isolate counts with strain # and antimicrobial tested (MIC).

MIC (µg/mL)	*E. coli*	*K. pneumoniae*	Others
Fig 1	EC25922 (N/A)		
Fig 2	EC69 CIP (≤0.25),<br>EC77 MEM (≤0.12),<br>EC55 MEM (>8)	KP79 CIP (>8)	
Fig 3	**EC1 GEN (4)**,<br>**EC85 MEM (1),**	**KP80 CIP (0.5)**	
Fig 4	**EC1 GEN (4)**,<br>EC69 CIP (≤0.0625),<br>EC85 CIP (>8),<br>**EC451 GEN (8),**<br>**EC543 GEN (16)**	KP126 CIP (0.125),<br>KP126 GEN (≤0.25),<br>**KP80 CIP (0.5),**<br>**KP76 CIP (1),**<br>KP79 GEN (>16)	
Fig 5	EC69 CIP (≤0.0625)		
Fig 6	**EC451 GEN (8)**		
Fig 7		**KP79 MEM (4)**	
Table 4	*Escherichia coli 2*<br>*Escherichia coli 4*<br>*Escherichia coli 16*	*Klebsiella pneumoniae 6*	*Citrobacter koseri 1*<br>*Enterobacter cloacae* complex *3*<br>*Serratia marcescens 5*<br>*Pseudomonas aeruginosa 7*<br>*Proteus penneri 8*<br>*Citrobacter koseri 9*

On-scale strains in bold.
