# Supplementary material for: Direct-from-specimen microbial growth inhibition spectrums under antibiotic exposure and comparison to conventional antimicrobial susceptibility testing
Source: PLoS One. 2022 Feb 16;17(2):e0263868. doi: 10.1371/journal.pone.0263868 (PMC8849476; doi:10.1371/journal.pone.0263868)
Supplement: S2 Table — A. GIC reporting values for Fig 3A. Ciprofloxacin GIC reporting with three algorithms for E. coli CDC 69 with a MIC of ≤ 0.0625 μg/mL and K. pneumoniae CDC 79 with an MIC of >8 μg/mL for Fig 3A. B. GIC reporting values for Fig 3B. Ciprofloxacin GIC reporting with three algorithms for E. coli CDC 69 with a MIC of ≤ 0.0625 μg/mL and K. pneumoniae CDC 79 with a MIC of >8 μg/mL for Fig 3B. C. GIC reporting values for Fig 3C. Meropenem GIC reporting with three algorithms for E. coli CDC 77 with a MIC of ≤ 0.12 μg/mL and E. coli CDC 55 with an MIC of > 8 μg/mL. (PDF) [file pone.0263868.s005.pdf]

S2A Table. GIC reporting values for Fig 3A.

**Sample**	Cutoff at GC = 0.4	Cutoff at GC = 0.5	Max. inhibition	GC signal (nA)	C0.0625 ratio	C0.125 ratio	C0.25 ratio	C0.5 ratio	C1 ratio	C2 ratio	C4 ratio
R strain, 30 min	>4	>4	>4	460	1.09	1.15	1.20	1.20	0.90	1.25	1.16
R strain, 60 min	>4	>4	>4	1065	1.07	1.07	1.33	1.31	1.11	1.16	1.04
R strain, 90 min	>4	>4	>4	3434	0.99	1.14	1.03	0.98	0.96	1.05	1.12
S strain, 30 min	>4	>4	2	847	1.10	0.91	0.94	0.98	0.74	0.54	0.57
S strain, 60 min	2	1	1	2845	0.73	0.80	0.70	0.62	0.48	0.28	0.23
S strain, 90 min	0.25	0.25	0.0625	6989	0.72	0.52	0.39	0.30	0.18	0.11	0.14

Ciprofloxacin GIC reporting with three algorithms for *E. coli* CDC 69 with a MIC of ≤ 0.0625 µg/mL and *K. pneumoniae* CDC 79 with an MIC of >8 µg/mL for Fig 3A.

S2B Table. GIC reporting values for Fig 3B.

Sample	Cutoff at GC = 0.4	Cutoff at GC = 0.5	Max. inhibition	GC signal (nA)	C0.0625 ratio	C0.125 ratio	C0.25 ratio	C0.5 ratio	C1 ratio	C2 ratio	C4 ratio
R strain, 30 min	>4	>4	>4	350	1.12	0.92	1.01	1.24	1.06	0.94	1.05
R strain, 60 min	>4	>4	>4	1121	0.94	0.96	0.97	1.03	1.13	0.81	0.81
R strain, 90 min	>4	>4	>4	2370	1.11	1.09	1.28	1.08	1.05	1.07	1.24
S strain, 30 min	>4	2	1	688	0.89	0.98	0.86	0.86	0.59	0.50	0.41
S strain, 60 min	1	1	1	1957	0.78	0.68	0.61	0.53	0.28	0.21	0.18
S strain, 90 min	0.25	0.125	≤0.0625	4853	0.62	0.46	0.40	0.26	0.18	0.09	0.04

Ciprofloxacin GIC reporting with three algorithms for *E. coli* CDC 69 with a MIC of ≤ 0.0625 µg/mL and *K. pneumoniae* CDC 79 with a MIC of >8 µg/mL for Fig 3B.

S2C Table. GIC reporting values for Fig 3C.

Sample	Cutoff at GC = 0.4	Cutoff at GC = 0.5	Max. inhibition	GC signal (nA)	M0.5 ratio	M1 ratio	M2 ratio	M4 ratio	M8 ratio	M16 ratio	M32 ratio
R strain, 1E6 CFU/mL	32	16	32	780	0.94	1.02	0.84	0.55	0.57	0.50	0.14
R strain, 1E7 CFU/mL	32	32	32	8065	1.00	1.00	1.00	0.92	0.79	0.62	0.17
R strain, 1E8 CFU/mL	>32	>32	>32	10000	1.00	1.00	1.00	1.00	1.00	1.00	1.00

S strain, 1E6 CFU/mL	1	1	1	968	0.73	0.40	0.30	0.16	0.03	0.02	0.02
S strain, 1E7 CFU/mL	2	2	≤0.5	9247	0.66	0.62	0.36	0.14	0.03	0.01	0.01
S strain 1E8 CFU/mL	16	16	16	10000	1.00	1.00	1.00	1.00	0.62	0.18	0.12

Meropenem GIC reporting with three algorithms for *E. coli* CDC 77 with a MIC of ≤ 0.12 μg/mL and *E. coli* CDC 55 with an MIC of > 8 μg/mL.
