# Supplementary material for: Direct-from-specimen microbial growth inhibition spectrums under antibiotic exposure and comparison to conventional antimicrobial susceptibility testing
Source: PLoS One. 2022 Feb 16;17(2):e0263868. doi: 10.1371/journal.pone.0263868 (PMC8849476; doi:10.1371/journal.pone.0263868)
Supplement: S3 Table — A. GIC reporting values for Fig 4A. Gentamicin GIC reporting with three algorithms for E. coli CDC 1 with an MIC of 4 μg/mL. B. GIC reporting values for Fig 4B. Meropenem GIC reporting with three algorithms for E. coli CDC 85 with an MIC of 1 μg/mL. C. GIC reporting values for Fig 4C. Ciprofloxacin GIC reporting with three algorithms for K. pneumoniae CDC 80 with an MIC of 0.5 μg/mL. D. GIC reporting values for Fig 4D. Ciprofloxacin GIC reporting with three algorithms for K. pneumoniae CDC 80 with an MIC of 0.5 μg/mL. (PDF) [file pone.0263868.s006.pdf]

S3A Table. GIC reporting values for Fig 4A.

Sample	Cutoff at GC = 0.4	Cutoff at GC = 0.5	Max. inhibition	GC signal (nA)	G1 ratio	G2 ratio	G4 ratio	G8 ratio	G16 ratio	G32 ratio
2 hours	4	4	2	3549	0.94	0.58	0.25	0.12	0.08	0.09
3 hours	2	2	2	5911	1.11	0.21	0.06	0.03	0.02	0.02
4 hours	2	2	2	9650	0.91	0.12	0.03	0.02	0.01	0.01

Gentamicin GIC reporting with three algorithms for *E. coli* CDC 1 with an MIC of 4 μg/mL.

S3B Table. GIC reporting values for Fig 4B.

Sample	Cutoff at GC = 0.4	Cutoff at GC = 0.5	Max. inhibition	GC signal (nA)	M0.5 ratio	M1 ratio	M2 ratio	M4 ratio	M8 ratio	M16 ratio	M32 ratio
2 hours	2	2	2	5669	0.84	0.80	0.37	0.35	0.02	0.01	0.01
3 hours	2	2	1	10000	0.97	0.52	0.14	0.02	0.01	0.00	0.00
4 hours	≤0.5	≤0.5	≤0.5	10000	0.18	0.06	0.03	0.01	0.01	0.00	0.00

Meropenem GIC reporting with three algorithms for *E. coli* CDC 85 with an MIC of 1 μg/mL.

S3C Table. GIC reporting values for Fig 4C.

Sample	Cutoff at GC = 0.4	Cutoff at GC = 0.5	Max. inhibition	GC signal (nA)	C0.0.625 ratio	C0.125 ratio	C0.25 ratio	C0.5 ratio	C1 ratio	C2 ratio	C4 ratio
2 hours	1	0.5	0.5	653	0.94	0.77	0.69	0.41	0.35	0.21	0.23
3 hours	0.125	0.125	0.125	5043	0.65	0.29	0.14	0.04	0.03	0.03	0.02
4 hours	0.125	0.125	0.125	9989	0.98	0.12	0.10	0.03	0.01	0.01	0.01

Ciprofloxacin GIC reporting with three algorithms for *K. pneumoniae* CDC 80 with an MIC of 0.5 μg/mL.

S3D Table. GIC reporting values for Fig 4D.

Sample	Cutoff at GC = 0.4	Cutoff at GC = 0.5	Max. inhibition	GC signal (nA)	C0.0.625 ratio	C0.125 ratio	C0.25 ratio	C0.5 ratio	C1 ratio	C2 ratio	C4 ratio
2 hours	1	0.5	0.5	564	1.04	0.97	0.78	0.50	0.27	0.23	0.19
3 hours	0.125	0.125	0.125	4411	0.71	0.25	0.12	0.07	0.04	0.03	0.03
4 hours	0.125	0.125	0.125	10000	0.86	0.10	0.04	0.02	0.01	0.01	0.01

Ciprofloxacin GIC reporting with three algorithms for *K. pneumoniae* CDC 80 with an MIC of 0.5 μg/mL.
