# Supplementary material for: Direct-from-specimen microbial growth inhibition spectrums under antibiotic exposure and comparison to conventional antimicrobial susceptibility testing
Source: PLoS One. 2022 Feb 16;17(2):e0263868. doi: 10.1371/journal.pone.0263868 (PMC8849476; doi:10.1371/journal.pone.0263868)
Supplement: S4 Table — A. GIC reporting values in three algorithms for Fig 5A. B. GIC reporting values in three algorithms for Fig 5B. (PDF) [file pone.0263868.s007.pdf]

S4A Table. GIC reporting values in three algorithms for Fig 5A.

Sample	Cutoff at GC = 0.4	Cutoff at GC = 0.5	Max. inhibition	GC signal (nA)	C0.0.625 ratio	C0.125 ratio	C0.25 ratio	C0.5 ratio	C1 ratio	C2 ratio	C4 ratio
EC69, MIC: ≤0.0625 μg/mL	≤0.0625	≤0.0625	≤0.0625	5163	0.18	0.06	0.06	0.04	0.03	0.02	0.01
KP126, MIC: 0.125 μg/mL	0.25	0.25	0.125	3591	0.96	0.56	0.35	0.20	0.11	0.08	0.08
KP80, MIC: 0.5 μg/mL	0.25	0.25	0.25	2622	0.91	0.60	0.28	0.16	0.08	0.08	0.06
KP76, MIC: 1 μg/mL	1	1	1	2596	0.92	0.90	0.98	0.86	0.30	0.17	0.11
EC85, MIC: >8 μg/mL	>4	>4	>4	2457	1.12	1.02	1.09	1.09	1.00	0.90	1.03

S4B Table. GIC reporting values in three algorithms for Fig 5B.

Sample	Cutoff at GC = 0.4	Cutoff at GC = 0.5	Max. inhibition	GC signal (nA)	G1 ratio	G2 ratio	G4 ratio	G8 ratio	G16 ratio	G32 ratio
KP126, MIC: ≤0.25 μg/mL	≤1	≤1	≤1	5763	0.01	0.00	0.00	0.00	0.00	0.00
EC1, MIC: 4 μg/mL	2	≤1	≤1	4339	0.43	0.07	0.02	0.01	0.01	0.01
EC451, MIC: 8 μg/mL	4	4	4	2614	0.93	0.59	0.13	0.01	0.01	0.01
EC543, MIC: 16 μg/mL	16	16	16	2776	1.45	1.32	1.07	0.80	0.18	0.02
KP79, MIC: >16 μg/mL	>32	>32	>32	2470	1.27	1.02	0.98	1.12	0.96	1.51
