# Supplementary material for: Direct-from-specimen microbial growth inhibition spectrums under antibiotic exposure and comparison to conventional antimicrobial susceptibility testing
Source: PLoS One. 2022 Feb 16;17(2):e0263868. doi: 10.1371/journal.pone.0263868 (PMC8849476; doi:10.1371/journal.pone.0263868)
Supplement: S5 Table — Ciprofloxacin GIC reporting with three algorithms for E. coli CDC 69 with a MIC of ≤ 0.0625 μg/mL. (PDF) [file pone.0263868.s008.pdf]

S5 Table. GIC reporting values for Fig 6.

Sample	Cutoff at GC = 0.4	Cutoff at GC = 0.5	Max. inhibition	GC signal (nA)	C0.0.625 ratio	C0.125 ratio	C0.25 ratio	C0.5 ratio	C1 ratio	C2 ratio	C4 ratio
1E5 CFU/mL – 1X	≤0.0625	≤0.0625	≤0.0625	444	0.14	0.11	0.10	0.07	0.08	0.07	0.06
1E5 CFU/mL – 0.1X	≤0.0625	≤0.0625	≤0.0625	77	0.25	0.36	0.34	0.27	0.22	0.22	0.30
1E6 CFU/mL – 1X	≤0.0625	≤0.0625	≤0.0625	7673	0.04	0.02	0.01	0.02	0.01	0.01	0.01
1E6 CFU/mL – 0.1X	≤0.0625	≤0.0625	≤0.0625	961	0.05	0.04	0.03	0.03	0.03	0.02	0.03
1E7 CFU/mL – 1X	0.125	0.125	≤0.0625	10000	0.59	0.21	0.15	0.09	0.06	0.04	0.02
1E7 CFU/mL – 0.1X	≤0.0625	≤0.0625	≤0.0625	10000	0.03	0.02	0.01	0.01	0.01	0.00	0.00
1E8 CFU/mL – 1X	2	2	1	10000	1.00	1.00	1.00	0.97	0.62	0.32	0.20
1E8 CFU/mL – 0.1X	0.125	0. 125	0.125	10000	0.88	0.28	0.19	0.13	0.07	0.04	0.03

Ciprofloxacin GIC reporting with three algorithms for *E. coli* CDC 69 with a MIC of ≤ 0.0625 μg/mL.
