# Supplementary material for: Direct-from-specimen microbial growth inhibition spectrums under antibiotic exposure and comparison to conventional antimicrobial susceptibility testing
Source: PLoS One. 2022 Feb 16;17(2):e0263868. doi: 10.1371/journal.pone.0263868 (PMC8849476; doi:10.1371/journal.pone.0263868)
Supplement: S6 Table — Gentamicin GIC reporting with three algorithms for E. coli CDC 451 with a MIC of 4 μg/mL. (PDF) [file pone.0263868.s009.pdf]

S6 Table. GIC reporting values for Fig 7.

Sample	Cutoff at GC = 0.4	Cutoff at GC = 0.5	Max. inhibition	GC signal (nA)	G1 ratio	G2 ratio	G4 ratio	G8 ratio	G16 ratio	G32 ratio
1E5 CFU/mL – 1X	4	≤1	≤1	248	0.49	0.58	0.19	0.05	0.06	0.05
1E5 CFU/mL – 0.1X	4	4	4	59	0.77	0.75	0.37	0.29	0.30	0.28
1E6 CFU/mL – 1X	4	4	4	2460	0.71	0.57	0.17	0.02	0.01	0.01
1E6 CFU/mL – 0.1X	4	4	4	326	0.84	0.77	0.21	0.06	0.06	0.05
1E7 CFU/mL – 1X	8	8	8	10000	1.00	1.00	0.95	0.05	0.02	0.02
1E7 CFU/mL – 0.1X	4	4	4	7199	0.95	0.66	0.14	0.01	0.01	0.00
1E8 CFU/mL – 1X	16	16	16	10000	1.00	1.00	1.00	1.00	0.31	0.21
1E8 CFU/mL – 0.1X	8	8	8	10000	1.00	1.00	1.00	0.09	0.04	0.02

Gentamicin GIC reporting with three algorithms for *E. coli* CDC 451 with a MIC of 4 μg/mL.
