# Supplementary material for: Direct-from-specimen microbial growth inhibition spectrums under antibiotic exposure and comparison to conventional antimicrobial susceptibility testing
Source: PLoS One. 2022 Feb 16;17(2):e0263868. doi: 10.1371/journal.pone.0263868 (PMC8849476; doi:10.1371/journal.pone.0263868)
Supplement: S7 Table — Meropenem GIC reporting with three algorithms for K. pneumoniae CDC 79 with a MIC of 4 μg/mL. (PDF) [file pone.0263868.s010.pdf]

S7 Table. GIC reporting values for Fig 8.

Sample	Cutoff at GC = 0.4	Cutoff at GC = 0.5	Max. inhibition	GC signal (nA)	M0.5 ratio	M1 ratio	M2 ratio	M4 ratio	M8 ratio	M16 ratio	M32 ratio
1E5 CFU/mL – 1X	4	4	4	147	1.30	1.08	1.05	0.16	0.14	0.08	0.08
1E5 CFU/mL – 0.1X	16	4	≤0.5	39	0.67	0.99	0.68	0.43	0.44	0.31	0.53
1E6 CFU/mL – 1X	4	4	4	2004	0.93	1.17	0.68	0.14	0.05	0.02	0.01
1E6 CFU/mL – 0.1X	4	4	4	237	0.86	0.94	0.66	0.22	0.17	0.12	0.12
1E7 CFU/mL – 1X	4	4	4	10000	1.00	1.00	1.00	0.20	0.11	0.04	0.03
1E7 CFU/mL – 0.1X	4	4	4	3604	0.99	1.00	0.62	0.10	0.06	0.04	0.03
1E8 CFU/mL – 1X	>32	>32	32	10000	1.00	1.00	1.00	1.00	1.00	1.00	0.51
1E8 CFU/mL – 0.1X	8	8	4	10000	1.00	1.00	1.00	0.62	0.31	0.15	0.12

Meropenem GIC reporting with three algorithms for *K. pneumoniae* CDC 79 with a MIC of 4 μg/mL.
