# Supplementary material for: Direct-from-specimen microbial growth inhibition spectrums under antibiotic exposure and comparison to conventional antimicrobial susceptibility testing
Source: PLoS One. 2022 Feb 16;17(2):e0263868. doi: 10.1371/journal.pone.0263868 (PMC8849476; doi:10.1371/journal.pone.0263868)
Supplement: S8 Table — (PDF) [file pone.0263868.s011.pdf]

S8 Table. GIC reporting of NYPQ blinded clinical specimens.

Sample NYPQ 1	Cutoff at GC = 0.4	Cutoff at GC = 0.5	Max. inhibition	GC signal (nA)	C0.0.625 ratio	C0.125 ratio	C0.25 ratio	C0.5 ratio	C1 ratio	C2 ratio	C4 ratio
1X	0.5	0.125	0.125	10000	0.80	0.50	0.47	0.32	0.22	0.06	0.04
0.1X	≤0.0625	≤0.0625	≤0.0625	7000	0.08	0.05	0.05	0.03	0.02	0.01	0.00

Sample NYPQ 2	Cutoff at GC = 0.4	Cutoff at GC = 0.5	Max. inhibition	GC signal (nA)	M0.5 ratio	M1 ratio	M2 ratio	M4 ratio	M8 ratio	M16 ratio	M32 ratio
1X	≤0.5	≤0.5	≤0.5	612	0.09	0.04	0.05	0.04	0.04	0.03	0.03
0.1X	1	≤0.5	≤0.5	65	0.49	0.27	0.20	0.18	0.21	0.18	0.16

Sample NYPQ 3	Cutoff at GC = 0.4	Cutoff at GC = 0.5	Max. inhibition	GC signal (nA)	G1 ratio	G2 ratio	G4 ratio	G8 ratio	G16 ratio	G32 ratio
1X	16	16	16	3384	0.78	0.85	1.04	0.80	0.13	0.04
0.1X	16	16	16	224	0.81	1.13	0.89	1.10	0.23	0.10

Sample NYPQ 4	Cutoff at GC = 0.4	Cutoff at GC = 0.5	Max. inhibition	GC signal (nA)	C0.0.625 ratio	C0.125 ratio	C0.25 ratio	C0.5 ratio	C1 ratio	C2 ratio	C4 ratio
1X	0.5	0.5	0.5	7861	1.04	0.88	0.89	0.25	0.06	0.03	0.02
0.1X	0.25	0.25	0.25	1071	0.72	0.54	0.21	0.10	0.05	0.03	0.02

Sample NYPQ 5	Cutoff at GC = 0.4	Cutoff at GC = 0.5	Max. inhibition	GC signal (nA)	M0.5 ratio	M1 ratio	M2 ratio	M4 ratio	M8 ratio	M16 ratio	M32 ratio
1X	4	1	≤0.5	642	0.55	0.48	0.42	0.23	0.16	0.12	0.09
0.1X	16	16	1	41	1.25	0.55	1.05	0.61	0.64	0.31	0.36

Sample NYPQ 6	Cutoff at GC = 0.4	Cutoff at GC = 0.5	Max. inhibition	GC signal (nA)	C0.0.625 ratio	C0.125 ratio	C0.25 ratio	C0.5 ratio	C1 ratio	C2 ratio	C4 ratio
1X	>4	>4	>4	10000	1.00	1.00	1.00	1.00	1.00	1.00	1.00
0.1X	>4	>4	>4	2484	1.05	0.80	0.89	0.66	0.77	0.89	0.80

Sample NYPQ 7	Cutoff at GC = 0.4	Cutoff at GC = 0.5	Max. inhibition	GC signal (nA)	M0.5 ratio	M1 ratio	M2 ratio	M4 ratio	M8 ratio	M16 ratio	M32 ratio
1X	≤0.5	≤0.5	≤0.5	310	0.07	0.07	0.06	0.05	0.08	0.07	0.07
0.1X	>32	32	≤0.5	34	0.60	0.51	0.62	0.68	0.51	0.61	0.46

Sample NYPQ 8	Cutoff at GC = 0.4	Cutoff at GC = 0.5	Max. inhibition	GC signal (nA)	G1 ratio	G2 ratio	G4 ratio	G8 ratio	G16 ratio	G32 ratio
1X	≤1	≤1	≤1	3748	0.05	0.03	0.02	0.02	0.02	0.01
0.1X	≤1	≤1	≤1	2406	0.02	0.02	0.01	0.01	0.01	0.01

Sample NYPQ 9	Cutoff at GC = 0.4	Cutoff at GC = 0.5	Max. inhibition	GC signal (nA)	G1 ratio	G2 ratio	G4 ratio	G8 ratio	G16 ratio	G32 ratio
1X	≤1	≤1	≤1	380	0.04	0.06	0.07	0.06	0.07	0.05
0.1X	2	2	≤1	53	0.59	0.33	0.33	0.25	0.34	0.32

Sample NYPQ 10	Cutoff at GC = 0.4	Cutoff at GC = 0.5	Max. inhibition	GC signal (nA)	G1 ratio	G2 ratio	G4 ratio	G8 ratio	G16 ratio	G32 ratio
1X	32	32	32	10000	1.00	1.00	1.00	1.00	1.00	0.28
0.1X	32	16	8	7778	1.06	0.94	1.08	0.59	0.43	0.03
