# Supplementary material for: Direct-from-specimen microbial growth inhibition spectrums under antibiotic exposure and comparison to conventional antimicrobial susceptibility testing
Source: PLoS One. 2022 Feb 16;17(2):e0263868. doi: 10.1371/journal.pone.0263868 (PMC8849476; doi:10.1371/journal.pone.0263868)
Supplement: S1 Protocol — (DOCX) [file pone.0263868.s012.docx]

**S9 Protocol - Molecular quantification of 16S rRNA with multiplexed-electrochemical biosensors through enzymatic amplification of redox current without using PCR**

**Introduction**

A variety of biosensors have been developed for the detection of biological material, such as pathogenic bacteria. The need for rapid detection as well as portability has led to the development of systems that couple pathogen recognition with signal transduction. Both optical and electrochemical detection of bacteria have been reported. However, the present electrochemical-based method has an advantage in that they are more amenable to miniaturization. The miniaturization of diagnostic tests offers many advantages over centralized laboratory testing, such as small reagent volumes, rapid analysis, small size and low power consumption, but should not compromise the ability to multiplex biomarkers, integrate multiple assays and achieve comparable clinical sensitivity and specificity. Here, we describe the detailed method for molecular quantification using functionalized electrochemical sensor that does not require nucleic acid amplification. This multiplex molecular-based on 16S rRNA sequence-specific hybridization, represents a sensitive, low-cost strategy for molecular diagnostics.

**Method overview**

The principle of this method is to convert the concentration of the target analyte such as 16S rRNA into an electrical current so the functionalized electrochemical sensor can precisely measure the electron flow that is proportional to the analyte concentration. Biosensors can be categorized by the reactant that they measure after the recognition event. Specifically, biosensor types include optical (light), bioluminescent (photons), thermal (heat), mass (resonance frequency changes), and electrochemical (electron transfer). While each of these types has inherent strengths and weaknesses, optical and electrochemical biosensors have become the most widely used. Optical biosensors have been adopted throughout clinical diagnostics and life science research due to their speed and sensitivity compared with other techniques. However, most optical biosensors still require the use of target amplification such that the signal is enhanced to a measurable level. Of these amplification methods, real-time PCR has emerged as the most widely adopted and is now considered the gold standard for the detection of nucleic acids from a variety of origins. Electrochemical biosensors promise superior speed and selectivity at a low cost. Historically, however, electrochemical biosensors lacked sufficient sensitivity for use beyond glucose monitoring and clinical chemistry analysis. Unlike previously reported electrochemical sensors that used graphite or carbon electrodes, a single layer of gold is used in this method for all three electrodes, i.e., working, auxiliary, and reference electrodes. Typically, Ag/AgCl or a saturated calomel electrode (SCE) is used as the reference electrode so that reversible oxidation/reduction occurs at a fixed potential at the reference electrode. In contrast, the Au is used as the reference electrode in this method because its properties of malleability and durability simplify fabrication and allow use of extremely thin electrodes. In this particular application, where the reduction of a mediator is monitored, Au can be successfully used as the reference electrode because a low voltage difference is maintained for short periods of time. The Au/Au/Au electrode system is characterized by cycling enzymatic amplification and targets are measured by amperometry.^^[[1]](#endnote-1)^^

*Chronoamperometry measurement*

Chronoamperometry involves stepping the bias potential at the working electrode from an initial potential to a final potential and holding that potential while the current is recorded at the electrode. These potentials are chosen so that they bracket the formal potential, E_0_, of the analyte. At the initial potential, no significant current flows. Once the potential is stepped to the final potential, the analyte is consumed at the electrode surface via oxidation or reduction (depending on the direction of the step). This depletes the concentration of the analyte near the electrode. The current response is thus a rapid increase followed by decay as the analyte is depleted and equilibrium is reached. The analysis of chronoamperometry or amperometry data is based on the Cottrell equation, which defines the current–time dependence for linear diffusion control. The final bias potential is determined such that the baseline current generated from electrolyte oxidation or reduction is minimized. This yields a better signal-to-noise ratio since the majority of background noise is from the electrolyte baseline. The Cottrell equation describes how the current, $i(t)$decays as a function of time, $t$

$i\left( t \right)={nFAC\left( \frac{D}{\pi} \right)}^{\frac{1}{2}}t^{-1/2}$, Equation 1 Cottrell equation

where $n$ is the number of electrons appearing in half-reaction for the redox couple, $F$ is Faraday’s constant (96,485 C/mol), $A$ the electrode area (cm^2^), $C$ the concentration of analyte (mole/L), $D$ the analyte’s diffusion coefficient (cm2/s), π = 3.14159, and $t$ is the time the current was measured (s). The current decays as the reciprocal of the square root of time. This dependence on the square root of time reflects the fact that physical diffusion is responsible for transport of the analyte to the electrode surface. The Cottrell plot is a straight-line graph plotted as $i (t)$ vs. $t^{-1/2}$ and can be used to determine concentration, the working electrode area or an analyte’s diffusion coefficient.

*Cycling enzymatic amplification using HRP and TMB*

Horseradish peroxidase (HRP) is one of the most widely used enzymes for analytical purposes because its high kinetic rate maximizes enzymatic signal amplification. By converting HRP-catalyzed electron transfer to an amperometric signal, the electrochemical sensor described in this method can effectively measure the number of HRP molecules immobilized on the sensor surface. Therefore, the output current is proportional to the number of molecular targets in the sample. The generalized reaction of peroxidases is an irreversible ping-pong mechanism that can be described by three sequential steps:

Step 1. The H_2_O_2_ in the substrate solution will first oxidize HRP into Compound I (${Fe}^{IV}=O^{-II}{(porphyrin)}^{\cdot+}$). HRP compound I is the oxidized form of HRP after losing two electrons. In other words, HRP will lose two electrons in the presence of H_2_O_2_, so it will be ready to react with TMB, which is the mediator.

$${Fe}^{III}\left( porphyrin \right)+H_{2}O_{2} \underset{\to}{k_{1}} {Fe}^{IV}=O^{-II}{(porphyrin)}^{\cdot+}+H_{2}O$$

Step 2. Oxidized HRP (Compound I) will oxidize the TMB (${AH}_{2}$) in the substrate solution and turn oxidized HRP (Compound I) into Compound II (${Fe}^{IV}=O^{-II}\left( porphyrin \right)H^{+}$). Concurrently the oxidized TMB ($AH^{\cdot}$) concentration will be increased and the amount will be proportional to the concentration of the analyte (16S rRNA) present. Neutral TMB is illustrated as AH_2_ and oxidized TMB is illustrated as $AH^{\cdot}$ in the following schematics.

$${Fe}^{IV}=O^{-II}\left( porphyrin \right)^{\cdot+}+{AH}_{2} \underset{\to}{k_{2}} {Fe}^{IV}=O^{-II}\left( porphyrin \right)H^{+}+AH^{\cdot}$$

Step 3. Compound II (${Fe}^{IV}=O^{-II}\left( porphyrin \right)H^{+}$)will also oxidize the TMB (${AH}_{2}$) and return to the original state of HRP. This step will not only increase the concentration of oxidized TMB ($AH^{\cdot}$) but also bring the HRP to its original state and ready to go back to Step 1 and react with H_2_O_2_ again. With sufficient TMB and H_2_O_2_, HRP can continuously be “recycled” and produce abundant oxidized TMB ($AH^{\cdot}$), which is the reactant to be measured by chronoamperometry.

$${Fe}^{IV}=O^{-II}\left( porphyrin \right)H^{+}+{AH}_{2} \underset{\to}{k_{3}} {Fe}^{III}\left( porphyrin \right)+AH^{\cdot}+H_{2}O$$

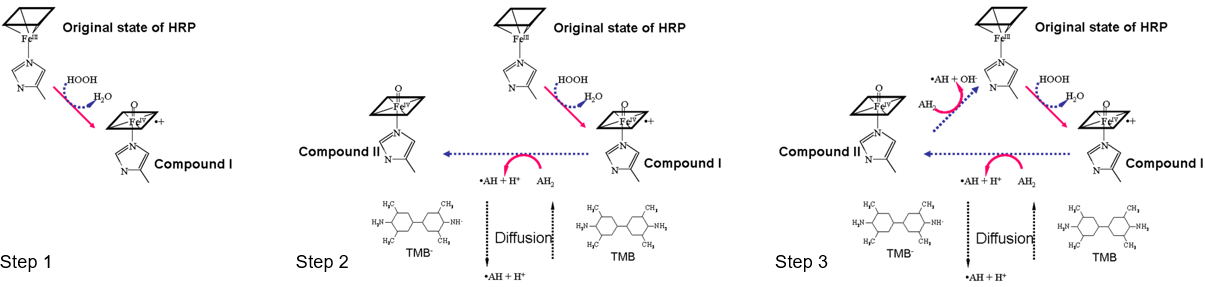


**Fig 1.** **Schematic representation of the HRP-TMB cycling enzymatic amplification**

*Electrochemical-based 16S rRNA measurement*

Electrochemical detection of 16S rRNA was performed as previously described for thiolated capture probes immobilized on photolithographically prepared Au electrode arrays, with modifications.^^[[2]](#endnote-2)^,^[[3]](#endnote-3)^,^[[4]](#endnote-4)^^ The detection strategy of the electrochemical-based sensors is based on sandwich hybridization of capture and detector oligonucleotide probes with target 16S rRNA in the lysate or biological samples. The capture probe is anchored to the gold sensor surface through 5’ end thiol conjugation, while the detector probe is conjugated with 3’ end fluorescein (FITC) conjugation for horseradish peroxidase (HRP) binding during the detection assay through anti-FITC antibody conjugated to the HRP. The sensor response was evaluated with a sandwich-type hybridization assay, using FITC as a tracer in the detector probe and anti-FITC-horseradish peroxidase (HRP) as the reporter molecule. 3,3’5,5’-tetramethylbenzidine (TMB)-H_2_O_2_ was the selected substrate for the electrochemical-based cycling enzymatic amplification measurement of the activity of the captured HRP reporter. The electrochemical sensor assay provides amperometric readout of the concentration of ribosomal RNA present in a sample. Capture and detector probes (or a probe pair) are designed to hybridize to species- and group-specific regions of the 16S rRNA molecule that are accessible to hybridization with oligonucleotide probes. Each sensor on the chip is functionalized with a specific pair of capture and detector probes.


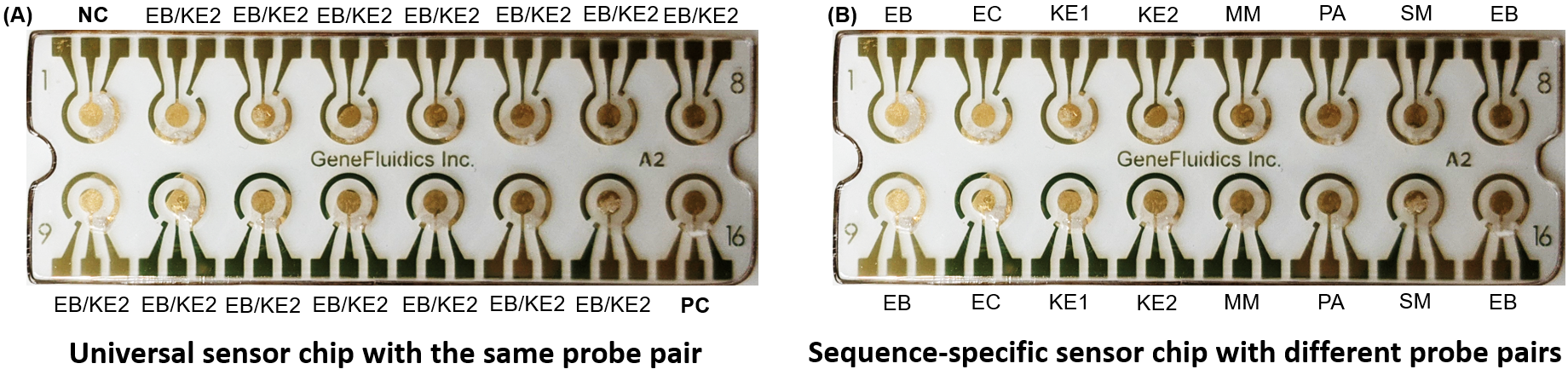


**Fig 2. Electrochemical sensor chip.** (A) Sensor configuration of using the same probe pair (EB/KE2 for most clinically relevant Gram-negative strains including *Enterobacterales* and *Pseudomonas aeruginosa*. EB capture probe: 5'-ACTTTATGAGGTCCGCTTGCTCT-3', EB detector probe: 5'-CGCGAGGTCGCCTTCCTTTGTAT-3', KE2 capture probe: 5'-GCACTTTATGAGGTCCGCTTGCTCT-3', KE2 detector probe: 5'-CGCGAGGTCGCTTCTCTTTGTATGC-3') for measurement of 16S rRNA from the same species with different conditions such as calibration curve, antimicrobial responses. (B) Sensor configuration of using different probe pairs for detection of 16S rRNA of an unknown sample such as in pathogen identification. NC stands for Negative Control and PC stands for Positive Control. Additional probe pairs (EC, KE1, MM, PA, SM) can be added for a customized panel for *E. coli*, *Pseudomonas aeruginosa*, *Serratia marcescens*, *Morganella morganii*, *Klebsiella pneumoniae*, *Klebsiella oxytoca*, *Enterobacter cloacae*, *Enterobacter aerogenes* and *Citrobacter freundii* to quantify 16S rRNA content individually.

When a substrate such as 3,3',5,5'-tetramethylbenzidine (TMB) is added to an electrode with capture target-detector complexes bound to its surface, the substrate is oxidized by HRP and reduced by the working electrode. This redox cycle results in shuttling of electrons by the substrate from the electrode to the HRP, producing enzymatic signal amplification of current flow in the electrode. The concentration of the target captured on the sensor surface can be quantified by the current obtained through the redox reaction between the TMB and HRP. Amperometric measurement of the catalyzed HRP reaction is obtained at a fixed potential of -200 mV applied by a potentiostat between the working and reference electrodes of all 16 sensors. Multiple sequence-specific genetic biomarkers can be quantified on the same sensor array chip with this universal detection approach.


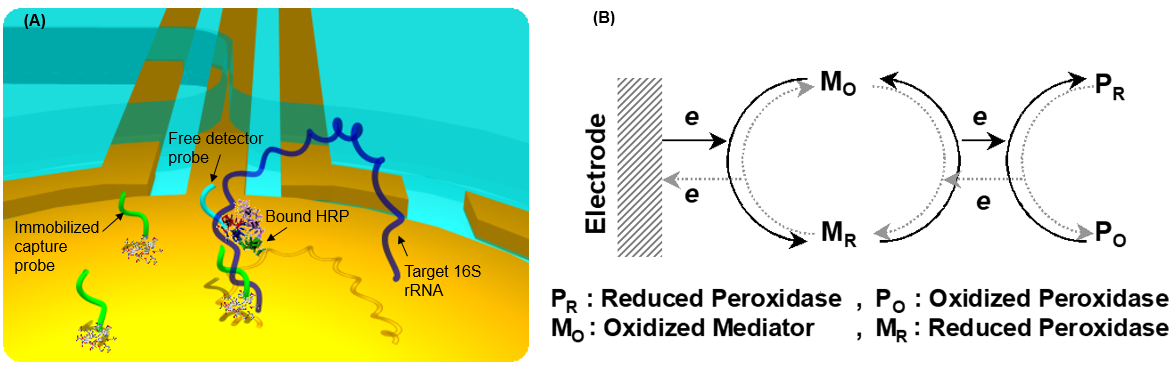


**Fig 3. Electrochemical detection of 16S rRNA.** (A) Sandwich-type hybridization of target RNA with capture and detection probes on the working electrode. (B) Signal transduction between the electrochemical sensor and the cyclic enzymatic amplification. Peroxidase is HRP and mediator is TMB in this method.

**Method details**

*Electrochemical sensor functionalization with sequence-specific oligonucleotide probe pairs*

Materials

- SC1000-16X-B bare gold electrochemical sensor array chip, GeneFluidics, Irwindale, CA
- Sequence-specific capture and detector probes and bridging oligonucleotide, Integrated DNA Technologies, Coralville, IA
- 1,6-Hexanedithiol 96% (HDT), Cat. No. H12005, Sigma Aldrich, St. Louis, MO
- 1x Casein Blocker in PBS, 1 Liter, Cat. No. 37528, Fisher Scientific, Waltham, MA
- Tris-EDTA: Cat. No. T9285, Sigma-Aldrich, St. Louis, MO
- MilliporeSigma™ Water, DEPC Treated, Sterile, Nuclease-Free, OmniPur™, Calbiochem™ (MilliporeSigma Cat. No. 9601100ML), Sigma Aldrich, St. Louis, MO
- 1X TE buffer is prepared by adding 0.5mL of Tris-EDTA into 49.5mL of DEPC water.
- Fish Gelatin Blocking Buffer, 10%, Cat. No. M319, VWR, Radnor, PA
- Tween 20, Cat. No. 9005-64-5, Fisher Scientific, Waltham, MA

**Protocol**

1. Dilute the stock capture probe with 1X TE buffer at a 1:99 ratio, then label it “Capture Probe”.

2. Dilute HDT with 1X TE buffer with ratio of 1.5:1000 and label it “HDT”

3. Add 25ul of “Capture Probe” and 15ul of “HDT” into 460ul of 1X Tris-EDTA buffer, mix well then wait for 10 minutes.

4. Pipet 6 μl onto the working electrode and incubate overnight (or at least 4 hours)

5. Following pre-cap incubation, wash off the pre-cap solution, deposit 6 μL of 1x Casein Blocker in PBS onto each sensor and incubate for 45 minutes.

6. During the incubation, make detector probe solution by adding stock detector probe into 0.75M PB at a 1:399 ratio. Then add Fish Gelatin Blocking Buffer at a 1:200 ratio and Tween 20 at a 1:1000 ratio.

7. Wash off the blocking agent, spot 6 μl per sensor onto working electrode, then air dry (~45 min) at 37°C.

8. During drying of the detector probe solution, make the EB/PA bridging oligo by diluting stock 100 µM EB1219 and KE2933 bridging oligonucleotide in a 1:2E5 ratio with Tris-EDTA buffer.

9. Once the detector probe has dried completely, add 6 μL of bridging oligo to sensor 16.

10. Collect the chips and store in a plastic mailer. Put the mailer into an anti-moisture bag with a pinch of desiccant and vacuum seal the bag. Store the chips in a desiccator.

**Protocol validation**

Establishing a limit of blank (LoB) for each specimen type followed by a quantitative calibration curve for limit of detection (LoD) determination is critical to ensure high positive predictive value (PPV) and negative predictive value (NPV). The LoB is the highest measured test result likely to be observed (typically at 95% certainty) for a sample containing no analyte. Figure 4 is an example from our *E.coli* detection validation study. The calculated LoB is 9.56 nA, which is very similar to the 95% percentile from the distribution plot in Figure 4A. LoD is the lowest concentration where analyte can be detected 95% of the time (a 5% likelihood of a false negative). Alternatively stated, LoD is the true value where the likelihood of a false negative measurement is 5%. The R^2^ value of the calibration curve fitting in Figure 4B is 0.973 and an accurate verification of bacteria count during calibration curve study is highly dependent on operator and methods.


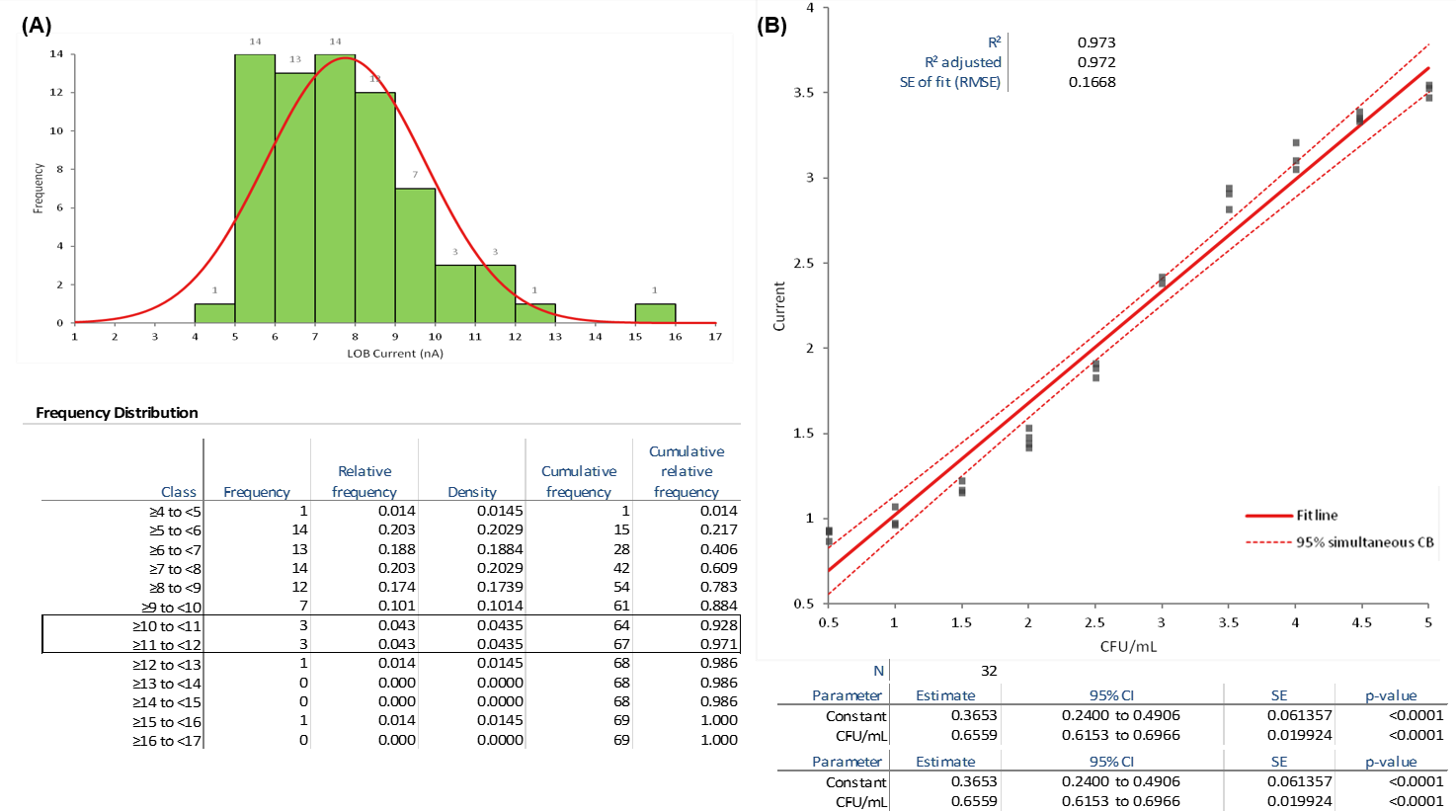


**Fig 4. Validation of electrochemical sensor detection.** (A) LoB frequency distribution plot by testing samples without target 16S rRNA content. (B) Calibration curve of *E.coli* spiked at various concentration (1-5 CFU/mL, N=32).

1. Gau V, Ma S-C, Wang H, Tsukuda J, Kibler J, Haake DA. Electrochemical molecular analysis without nucleic acid amplification. Methods. 2005;37(1): 73-83. doi: 10.1016/j.ymeth.2005.05.008. [↑](#endnote-ref-1)
2. Gau JJ, Lan EH, Dunn B, Ho CM, Woo JC. A MEMS based amperometric detector for E. coli bacteria using self-assembled monolayers. Biosens Bioelectron. 2001;16(9-12): 745-55. doi: 10.1016/s0956-5663(01)00216-0. [↑](#endnote-ref-2)
3. Sun C-P, Liao JC, Zhang Y-H, Gau V, Mastali M, Babbitt JT, et al. Rapid, species-specific detection of uropathogen 16S rDNA and rRNA at ambient temperature by dot-blot hybridization and an electrochemical sensor array. Mol Genet Metab. 2005;84(1): 90-9. doi: 10.1016/j.ymgme.2004.11.006. [↑](#endnote-ref-3)
4. Mach KE, Mohan R, Baron EJ, Shih M-C, Gau V, Wong PK, et al. A Biosensor Platform for Rapid Antimicrobial Susceptibility Testing Directly from Clinical Samples. J Urol. 2011;185(1): 148-153. doi: 10.1016/j.juro.2010.09.022. [↑](#endnote-ref-4)
