# Supplementary material for: Direct-from-specimen microbial growth inhibition spectrums under antibiotic exposure and comparison to conventional antimicrobial susceptibility testing
Source: PLoS One. 2022 Feb 16;17(2):e0263868. doi: 10.1371/journal.pone.0263868 (PMC8849476; doi:10.1371/journal.pone.0263868)
Supplement: Attachment — Submitted filename: Plos.docx [file pone.0263868.s013.docx]

The manuscript “Direct-from-specimen microbial growth inhibition spectrums under antibiotic exposure and comparison to conventional antimicrobial susceptibility testing” proposes an antimicrobial efficacy profiling method that can be performed in hours directly from clinical urine specimens. The study seems very interesting, and clearly advances the current science. Although authors have worked on an interesting area of research, the figure quality is too poor to judge the manuscript. I am unable to read anything; the printing was also not helpful. Please submit higher resolution images for proper review. Please avoid abbreviations in the abstract. Also, adding a graphical abstract will be helpful for the readers of Plos one.
