# Supplementary material for: Direct-from-specimen microbial growth inhibition spectrums under antibiotic exposure and comparison to conventional antimicrobial susceptibility testing
Source: PLoS One. 2022 Feb 16;17(2):e0263868. doi: 10.1371/journal.pone.0263868 (PMC8849476; doi:10.1371/journal.pone.0263868)
Supplement: Attachment — Submitted filename: Response to Reviewers.docx [file pone.0263868.s014.docx]

Response to Reviewers

PONE-D-21-08657

Dear Editor,

We would like to thank you and the reviewers for taking the time to consider our work and provide feedback. We appreciate the reviewers’ detailed feedback, which has proved to be helpful in improving the quality of our manuscript. We have adjusted our manuscript accordingly and addressed each comment in italics with references to the clean copy of the revised manuscript.

Journal Requirements:

When submitting your revision, we need you to address these additional requirements.

1. Please ensure that your manuscript meets PLOS ONE's style requirements, including those for file naming. The PLOS ONE style templates can be found at

<https://journals.plos.org/plosone/s/file?id=wjVg/PLOSOne_formatting_sample_main_body.pdf> and

[https://journals.plos.org/plosone/s/file?id=ba62/PLOSOne_formatting_sample_title_authors_affiliations.pdf](about:blank)

*Response: We have ensured our manuscript meets journal requirements: figure and table references, figure and table captions and legends, section headings, etc.*

2. We noticed you have some minor occurrence of overlapping text with the following previous publication(s), which needs to be addressed:

- <https://journals.asm.org/doi/10.1128/jcm.01999-17?permanently=true>

The text that needs to be addressed involves the second paragraph of the Discussions section.

In your revision ensure you cite all your sources (including your own works), and quote or rephrase any duplicated text outside the methods section. Further consideration is dependent on these concerns being addressed.

*Response: We have amended lines 482-490 to address the overlapping text. We have also checked all citations for other instances of overlapping text.*

**Comments to the Author**

1. Is the manuscript technically sound, and do the data support the conclusions?

The manuscript must describe a technically sound piece of scientific research with data that supports the conclusions. Experiments must have been conducted rigorously, with appropriate controls, replication, and sample sizes. The conclusions must be drawn appropriately based on the data presented.

Reviewer #1: Partly

Reviewer #2: Partly

*Response:* *We have added the number of data points for each graph in each figure caption. We have also revised the statistical analysis section (line 170) to provide more details.*

2. Has the statistical analysis been performed appropriately and rigorously?

Reviewer #1: No

Reviewer #2: N/A

*Response:* *We have added the number of data points for each graph in each figure caption. We have also revised the statistical analysis section (line 170) to provide more details.*

3. Have the authors made all data underlying the findings in their manuscript fully available?

The [PLOS Data policy](http://www.plosone.org/static/policies.action#sharing) requires authors to make all data underlying the findings described in their manuscript fully available without restriction, with rare exception (please refer to the Data Availability Statement in the manuscript PDF file). The data should be provided as part of the manuscript or its supporting information, or deposited to a public repository. For example, in addition to summary statistics, the data points behind means, medians and variance measures should be available. If there are restrictions on publicly sharing data—e.g. participant privacy or use of data from a third party—those must be specified.

Reviewer #1: Yes

Reviewer #2: Yes

4. Is the manuscript presented in an intelligible fashion and written in standard English?

PLOS ONE does not copyedit accepted manuscripts, so the language in submitted articles must be clear, correct, and unambiguous. Any typographical or grammatical errors should be corrected at revision, so please note any specific errors here.

Reviewer #1: No

Reviewer #2: Yes

*Response: We have made changes throughout the manuscript to more clearly present our research findings and enhance the flow of content.*

5. Review Comments to the Author

Please use the space provided to explain your answers to the questions above. You may also include additional comments for the author, including concerns about dual publication, research ethics, or publication ethics. (Please upload your review as an attachment if it exceeds 20,000 characters)

Reviewer #1: Summary of the manuscript:
I would like to appreciate the authors for their efforts, overall ideology and execution of the work is commendable. But language of the manuscript is not upto the mark, it has lots of grammatical errors and improper sentence formations. The manuscript needs an extensive review of the English language, hence this manuscript can’t be considered for publication in the same format.

*Response: We have made changes throughout the manuscript to more clearly present our research findings and enhance the flow of content.*

Specific corrections
Avoid general statements, results and discussions in the methodology part, it can be included in respective sections of the manuscript.

*Response: We have removed such statements from the methods section.*

Authors may avoid some unsound words in manuscript such as, very, see etc.

*Response: We have made changes throughout the manuscript to address this concern.*

Authors may provide figures with high clarity

*Response: We have improved the quality of the figures.*

Reviewer #2: The manuscript “Direct-from-specimen microbial growth inhibition spectrums under antibiotic exposure and comparison to conventional antimicrobial susceptibility testing” proposes an antimicrobial efficacy profiling method that can be performed in hours directly from clinical urine specimens. The study seems very interesting, and clearly advances the current science. Although authors have worked on an interesting area of research, the figure quality is too poor to judge the manuscript. I am unable to read anything; the printing was also not helpful. Please submit higher resolution images for proper review. Please avoid abbreviations in the abstract. Also, adding a graphical abstract will be helpful for the readers of Plos one.

*Response: We have removed all abbreviations in the abstract. We have improved the quality of the figures and the surrounding text. We have included a graphical abstract (Fig 1) illustrating the presented method in line 76.*
