# Supplementary material for: Direct-from-specimen microbial growth inhibition spectrums under antibiotic exposure and comparison to conventional antimicrobial susceptibility testing
Source: PLoS One. 2022 Feb 16;17(2):e0263868. doi: 10.1371/journal.pone.0263868 (PMC8849476; doi:10.1371/journal.pone.0263868)
Supplement: Attachment — Submitted filename: Response to Reviewers.docx [file pone.0263868.s015.docx]

Response to Reviewers

PONE-D-21-08657R1

Dear Editor,

We would like to thank you and the reviewers for considering our manuscript for publication. We appreciate the time and effort taken to provide feedback. Although comments were minor, we believe they were helpful to ensure the best version of our manuscript. We have addressed all comments below in italics and have referenced the manuscript with tracked changes in our responses.

Reviewers' comments:

Reviewer's Responses to Questions

Comments to the Author

1. If the authors have adequately addressed your comments raised in a previous round of review and you feel that this manuscript is now acceptable for publication, you may indicate that here to bypass the “Comments to the Author” section, enter your conflict of interest statement in the “Confidential to Editor” section, and submit your "Accept" recommendation.

Reviewer #1: All comments have been addressed

Reviewer #2: (No Response)

2. Is the manuscript technically sound, and do the data support the conclusions?

The manuscript must describe a technically sound piece of scientific research with data that supports the conclusions. Experiments must have been conducted rigorously, with appropriate controls, replication, and sample sizes. The conclusions must be drawn appropriately based on the data presented.

Reviewer #1: Yes

Reviewer #2: Partly

***Response:*** *We have added statistical analysis in the supporting information. Please see response below.*

3. Has the statistical analysis been performed appropriately and rigorously?

Reviewer #1: Yes

Reviewer #2: No

***Response:*** *We have added statistical analysis in the supporting information. Please see response below.*

4. Have the authors made all data underlying the findings in their manuscript fully available?

The [PLOS Data policy](http://www.plosone.org/static/policies.action#sharing) requires authors to make all data underlying the findings described in their manuscript fully available without restriction, with rare exception (please refer to the Data Availability Statement in the manuscript PDF file). The data should be provided as part of the manuscript or its supporting information, or deposited to a public repository. For example, in addition to summary statistics, the data points behind means, medians and variance measures should be available. If there are restrictions on publicly sharing data—e.g. participant privacy or use of data from a third party—those must be specified.

Reviewer #1: Yes

Reviewer #2: Yes

5. Is the manuscript presented in an intelligible fashion and written in standard English?

PLOS ONE does not copyedit accepted manuscripts, so the language in submitted articles must be clear, correct, and unambiguous. Any typographical or grammatical errors should be corrected at revision, so please note any specific errors here.

Reviewer #1: Yes

Reviewer #2: Yes

6. Review Comments to the Author

Please use the space provided to explain your answers to the questions above. You may also include additional comments for the author, including concerns about dual publication, research ethics, or publication ethics. (Please upload your review as an attachment if it exceeds 20,000 characters)

Reviewer #1: All the comments have been addressed by authors, I would like to recommend this manuscript for the publication.

***Response:*** *We thank you for your time and support.*

Reviewer #2: The paragraph “Even though antibiotics do not directly affect the SARS-CoV-2 respiratory virus responsible for the COVID-19 48 pandemics, physicians are administering many more antibiotics than normal when treating COVID-19 patients 49 [2]. As published in the New England Journal of Medicine, 58.0% of the surveyed 1,099 COVID-19 patients 50 received intravenous antibiotic therapy in China, whereas 35.8% received oseltamivir antiviral therapy [3]. The 51 appearing surge in antibiotic use is reflected in the higher percentages of COVID-19 patients with severe 52 conditions and pediatric patients (85% in a multicenter pediatric COVID-19 study [4]) receiving antibiotic 53 therapies. The World Health Organization warned that the use of antibiotic therapy may lead to higher bacterial 54 resistance rates and increase the burden of the pandemic [5]. As viral respiratory infections often lead to bacterial 55 pneumonia, physicians may struggle to identify the causative pathogen in lung infections. A recent study by Zhou 56 et al. [6] found that 15% of 191 hospitalized COVID-19 patients, as well as 50% of the 54 non-survivors, acquired 57 bacterial infections. Major outbreaks of other respiratory viruses illustrated the same concern. For example, the 58 majority of deaths from the 1918 flu showed autopsy results consistent with bacterial pneumonia, and up to half 59 of the 300,000 non-survivors of the 2009 H1N1 flu were confirmed to have died from pneumonia [7-8]. Therefore, 60 a shorter time to rule out certain antibiotic options by detecting microbial growth under such conditions may 61 provide physicians with valuable information before the availability of conventional AST results,” contains some irrelevant information and it must be shortened.

***Response****: We have removed irrelevant information and shortened the paragraph in lines 47-61.*

Where statistical analyses are described, please specify what formal tests for normality were used to assess data distribution. All data should be subject to tests for normality. Data that do not exhibit a normal/Gaussian distribution should be analyzed via a non-parametric equivalent.

**Response:** *The main premise of this paper is to demonstrate the feasibility of a direct-from-specimen AST that does not require clinical isolates as most current FDA-approved and CLSI methods do. A larger sample size for testing of clinical samples would be our next step to further validate the clinical utility of the presented method, and the normality test would then be essential for that future study. However, we have taken your suggestion and added a paragraph to the statistical analysis section (lines 205-211) to explain our normality tests for the negative and growth controls. Additionally, we reference and briefly explain each supplemental figure in lines 251-255, 282-284, and 317-322.*

*In the future study using a larger sample size of blinded clinical samples, the microbial load distribution would not be predictable and therefore not normal, as samples could be negative, high positive, or weak positive (>80% of culture samples are reported negative).*

7. PLOS authors have the option to publish the peer review history of their article ([what does this mean?](https://journals.plos.org/plosone/s/editorial-and-peer-review-process#loc-peer-review-history)). If published, this will include your full peer review and any attached files.


If you choose “no”, your identity will remain anonymous but your review may still be made public.


Do you want your identity to be public for this peer review? For information about this choice, including consent withdrawal, please see our [Privacy Policy](https://www.plos.org/privacy-policy).

Reviewer #1: No

Reviewer #2: No
